# No Country for Oldowan Men: Emerging Factors in Language Evolution

**DOI:** 10.3389/fpsyg.2019.01448

**Published:** 2019-06-19

**Authors:** Elliot Murphy

**Affiliations:** Division of Psychology and Language Sciences, University College London, London, United Kingdom

**Keywords:** domestication syndrome, molecular clock, genetic drift, globularity, language evolution, basicranial angle, birdsong

## Abstract

Language evolution has long been researched. I will review a number of broad, emerging research directions which arguably have the potential to contribute to our understanding of language evolution. Emerging topics in genomics and neurolinguistics are explored, and human-specific levels of braincase globularity – and the broader process of self-domestication within which globularity seems capable of being encapsulated – will be argued to be the central pillars of any satisfactory and interdisciplinary model of language evolution.

## Introduction

In recent years, a number of models have been proposed to explain the implementational basis of hierarchical phrase structures (reviewed in Aboitiz, 2017; Friederici, 2017). A range of paleoanthropological, paleoneurological and genetic data has also been consulted in an effort to map out an accurate path that language evolution likely took (Zollikofer and Ponce de León, 2013; Benítez-Burraco and Boeckx, 2015; Beaudet, 2017; Murphy and Benítez-Burraco, 2018a, b). My intention here is to review some possible connections between these distinct modes of inquiry by exploring a specific set of phenotypic traits and evolutionary processes which have the potential to explain the emergence of core features of language such as syntactic complexity and unrestricted semantic combinatorics.

This review will begin by focusing on genetics (“Gene Regulation,” “Genetic Drift,” “Comparative Genomics,” “Molecular Clock,” and “DNA Sequencing”) and then progress to broader evolutionary themes (“Globularity,” “Tool Use”) and emerging directions (“Domestication,” “The Cerebellum and Speech”).

## Gene Regulation

Beginning with the genetic foundation of a possible model of language evolution, we can consider what the likely *mutational profile* of its initial stages were. It has been proposed that there exist 1,241 primate-specific genes (Zhang et al., 2011), 280 of which are human-specific. Fifty-four percent of these human-specific genes are upregulated in a brain area implicated in higher cognition, the prefrontal cortex. These new genes are significantly more likely to be involved in gene regulation (Diller and Cann, 2013, p. 256), although, as we will see below, exploring the genetic basis of other brain regions will also be required to account for language evolution. The mutation of some regulatory gene may have reorganized the neuronal populations in the neocortex and its concomitant computational properties (although the precise nature of these neurolinguistic properties is beyond the scope of this general review; see Friederici, 2017). Given the level of regulatory complexity identified by Chakravarti (2011) – “compromising the activity of one gene need not cripple an entire network”; “variation in the regulatory machinery of genes is much more frequent than that in the structure of gene products” – it is more likely that the neurocomputational properties required for language emerged after the mutation of multiple regulatory genes acting in concert, and not a singular mutational event as often claimed in the generative and biolinguistics literature (e.g., Chomsky, 2010): “Genes and their products almost never act alone, but in networks with other genes and proteins and in [the] context of the environment” (Chakravarti, 2011, p. 15).

Is there any indication that this general picture is reasonable? Consider how the transition from the many digits of lobe-finned tetrapods to only 5 was not the result of new genes, but rather of distinct regulations of existing genes, namely regulation of *Hoxa11* (Kherdjemil et al., 2016). A similar account may be applied to core features of language, in particular given that there exists no strong correlation between the total number of genes in a given species and the level of biological complexity it achieves (for instance, mice and humans have comparable numbers). Of relevance here is the finding that human evolution has slowed down, often called the “hominoid slowdown”: “[R]ates of occurrence of *de novo* mutations decreased as enhanced DNA repair mechanisms and larger generation times evolved” (Goodman, 1985, p. 10). Hominoids appear to have reached a certain mesa of complexity, with only slight tuning yielding novel benefits.

In summary, a slight regulatory change could have produced an alteration in the human computational system yielding the capacity for constructing hierarchical phrase structures.

A separate question now concerns when this took place. Putting aside precise dates, and assuming that anatomically modern humans emerged around 300–150 kya (kya: 1000 years ago), it appears that the vast majority of complex forms of symbolic representation did not emerge until 100–60 kya (Hurford, 2011). We will present a more detailed timeline below after considering a broader range of topics, but for now we can note that this time also correlates with the emergence of new migration patterns (Mellars, 2006), leading to the possibility that properties of the environment acted as release factors for language. Encountering new forms of social organization and environments may have served to prompt the basic combinatorics of mammalian cognition and encourage novel forms of conceptual combination. Bolender (2007) has suggested along these lines that an increased human population, leading to a greater complexity of inter-group communication, acted as a trigger for the use of syntactic word movement, hitherto dormant. If this is correct, then investigating syntactic phenomena from a purely computational perspective, not considering the influence of the development and emergence of the phenotype, would be missing a crucial part of any psychological or cognitive account.

## Genetic Drift

We can now turn to a related topic, which has become just as controversial in the language evolution literature. One of the most general distinctions in this literature is between theories that assume language emerged suddenly, and theories that assume it emerged gradually. What does the archeological record have to say about this? Unfortunately, since the African middle Pleistocene hominin record is sparse, it is currently not possible to determine whether fossils like Omo Kibish 1 mark the earliest forms of the constellation of human features or whether older types exist. Another major question concerns whether human features emerged through natural selection or through random *genetic drift*. This occurs when the proportion of a gene variant in a population changes due to external events (“chance”). Coyne (2009, p. 14) notes that “genetic drift may play some evolutionary role in small populations and probably accounts for some non-adaptive features of DNA.” Examining cranial measurements, Weaver et al. (2008) show that the differences between Neanderthals and anatomically modern humans could have emerged under drift over a period of around 400,000 years. Moreover, Weaver and Stringer (2015) show that these cranial differences emerged in a highly unconstrained way thanks to cultural buffering, relative to morphological divergences documented between crania of subspecies of *Pan troglodytes*.

While it is well known that only ∼4% of the human genome differs at the nucleotide level from the chimpanzee genome (Varki and Altheide, 2005), the way that these genes are expressed is far from uniform. For instance, there is up to an 8% difference in splicing rates in the cortex between humans and chimpanzees (Calarco et al., 2007), with NDE1 (a gene involved in cortical neurogenesis) recently being shown to exhibit human-specific splicing patterns. Splicing consequently seems to be a major mechanism of brain evolution and cognitive development (Mosca et al., 2017).

## Comparative Genomics

Comparative genomics yields other fruitful insights into the likely origins of language. Gronau et al. (2011) analyzed the whole-genome variation diversity patterns of six people from contemporary sub-populations: European, Yoruban, Han Chinese, Korean, Bantu, and San African. The final group (speakers of Khoisan) were discovered to have likely split from the rest of the human population around 157–108 kya, and since they possess the ability to acquire language this indicates a likely timeline. Behar et al. (2008) report that mitochondrial DNA (mtDNA, transmitted through maternal inheritance) in the Khoisan peoples diverged from mtDNA in the human gene pool as early as 160 kya years ago, remaining separate until around 40 kya. The genetic isolation of the San people matches with the isolation of a core part of their language use. All Khoisan groups use clicks; Moisik and Dediu (2017) use a biomechanical model to show that a reduced alveolar ridge aids the production of clicks, and that this has been selected for amongst Khoisan groups. Clicks are complex obstruents externalized via a double closure in the oral cavity. Huybregts (2017) notes the intriguing possibility which follows from these findings. The common human population shared by the San and the rest of contemporary human societies must have had language but may not have solved the problem of externalization, i.e., they may have exhibited the ability to recursively construct hierarchical representations, but not the ability to map this capacity to the sensorimotor system for externalization via speech, gesture, and so forth. The San population and the non-San populations therefore solved the problem in different ways, indicating a clear timeline: the computational system of language evolved before it was linked to externalization.

Nielsen et al. (2017) also discuss how “genetic markers with uniparental inheritance and linguistic studies suggest that click-language-speaking hunter-gatherer populations may originally have been more widespread and were replaced in areas other than southern Africa or, alternatively, that they may have originated in eastern Africa and then migrated to southern Africa in the past 50 kyr.” Furthermore, “other hunter-gatherer populations that speak languages that use clicks, including the Hadza people and the Sandawe people, currently reside in Tanzania in eastern Africa, although they display limited genomic affinity with the San people of southern African.”

Lastly, despite the question of modern human origins in Africa remaining unsettled, a multiregional origin in which modern (domesticated) features evolved in a fragmented way in multiple areas connected by gene flow is a strong possibility. There is evidence, for instance, for the admixture of modern humans with archaic populations in Africa (Hammer et al., 2011). Statistical analyses of whole-genome sequencing data from geographically diverse hunter-gatherer populations also presents evidence of archaic human lineages that underwent introgression (i.e., exchanging genetic material via interbreeding) and diverged from modern human lineages anywhere between 1.3 mya and 35 kya, and so the extent of archaic admixture remains a point of controversy: “Perhaps of greatest interest is genomic data from under-sampled regions of the world, which may help to refine evolutionary theories, including the question of whether there are further, as-yet uncharacterized, lineages of archaic humans” (Nielsen et al., 2017, p. 308).

## Molecular Clock

Another topic which I would like to argue is relevant for language evolution research is the *molecular clock*, in particular given that many core hypotheses about the origin of recursive hierarchical phrase structure concern sudden and chance mutations. In recent research, the speed of the molecular clock has been calculated in terms of the number of mutational differences in matching segments of DNA between humans and primates based on the fossil record. Because it has typically been assumed that the speed was high, the “Out of Africa” migration was thought to have occurred around 70 kya (e.g., Gibbons, 2012). More recently, however, a new method of obtaining mutation rates has emerged which calculates the rate of the full genome of present-day humans through counting the number of new mutations in the nuclear DNA of a newborn compared to its parents. Scally and Durbin (2012) cite the value at 0.5 × 10^–9^ bp^–1^ year^–1^, which is around half of the previous fossil-calibrated rate (Ike-uchi, 2016).

As such, the molecular clock is much slower than previously believed. Adjusting for these new calculations, the migration from Africa is likely to have occurred around 130 kya (Ike-uchi, 2016) (as the fossil record also suggests).

A possible scenario for language evolution in line with these findings is that the mutation(s) required for language occurred in an individual between 200 and 130 kya in East Africa. This then spread through the community, and around 130 kya a group (composed of around ∼450 individuals, according to estimates in Fagundes et al., 2007) migrated north across Arabia, passing the Bab al-Mandab Straits and progressing to Oman and the surrounding regions, eventually arriving in southern China and Indo-China. A separate group, much later (100–50 kya) also left North Africa through a different route (the Nile Valley) and reached Eurasia. Of course, the hypothesis that a small number of mutations in a relatively short time window led to language is naturally compatible with whatever theory one adopts concerning the speed (fast or slow) of the molecular clock. But the notion of a slow clock nevertheless makes the standard generative picture of a *sudden, slight mutation* somewhat less appealing, and rather points to the validity of a series of mutations. None of these discrete changes would have likely been sufficient to bring about the morphological and neurological characteristics of the anatomically modern human brain, but when spread throughout a community for extended periods they may have conspired to do so.

## DNA Sequencing

Having covered some broad topics in genomics, what can be said about the emerging theme of technological advances with potential to inform models of language evolution? Developments in DNA sequencing recently resulted in sequence data covering much of the Neanderthal genome (Green et al., 2010). Shortly thereafter, a list of 87 genes with protein-coding differences between humans and Neanderthals was released (Prüfer et al., 2014). This allows hypotheses to be drawn up concerning the existence of certain language-relevant cognitive components in Neanderthals. The most famous (and notorious) candidate for a “language gene” is *FOXP2*. This codes for a transcription factor (a protein able to bind DNA and modify the expression of other genes) connected to a large network of genes that can be up- or down-regulated (Vernes et al., 2007). In modern humans the gene exists in a species-specific allele, coding a protein differing from that of chimpanzees (Enard et al., 2002). *FOXP2* currently seems to have no variation that might have distinguished Neanderthals/Denisovans from humans. But as DeSalle and Tattersall (2017) note, this is an extremely weak basis from which to claim that Neanderthals/Denisovans had language. Prüfer et al. (2014) drew up a list of candidates for the Neanderthal genome and, as DeSalle and Tattersall (2017, p. 5) comment, these authors “do not appear to have made any strong connections between language and any of the genes they determined as important in the differentiation of the Neanderthal/Denisovan genomes.” Of all the candidate genes for language summated via extensive review by DeSalle and Tattersall (2017), only one has a serious and promising connection to the Prüfer et al. (2014) database: CNTNAP2. This plays an important role in nervous system development and covers 1.5% of chromosome 7, although it currently remains unclear how it could causally relate to language evolution (see Mountford and Newbury, 2018 for further discussion). A regulatory region of FOXP2 was recently identified exclusively in modern humans at a binding site of the transcription factor POU3F2 (Maricic et al., 2013). This documented POU3F2 change that enhanced FOXP2 expression in the human brain was also not part of the gene flow from humans into Neanderthals that occurred in the Levant or Southern Arabia 125–100 kya (Kuhlwilm et al., 2016). Since this likely resulted in improved speech, it is not unreasonable to associate linguistic externalization with this POU3F2 haplotype at FOXP2, suggesting that externalization was a late development occurring after the initial computational system had emerged. This research suggests that “differences in gene regulation and expression may be involved in cognitive function, and that species differences are due to far more than just two variants in a single gene” (Mountford and Newbury, 2018, p. 55).

Building on these developments, Murphy and Benítez-Burraco (2018b) argue that since we cannot track the neuronal activity of the brain from extinct hominins, it is reasonable to use our current understanding of the language “oscillogenome” (that is, the set of genes responsible for basic aspects of oscillatory brain activity relevant for language; see Murphy and Benítez-Burraco, 2018a) to infer some properties of the Neanderthal oscillatory profile. Several candidates for the language oscillogenome show differences in their methylation patterns between Neanderthals and humans, and Murphy and Benítez-Burraco (2018b) claim that differences in their expression levels could be informative of differences in cognitive functions important for language (e.g., working memory).

Exploring a broad topic such as the genetics of language will require a number of linking hypotheses between genes, neural anatomy and cognitive processes. Without such linking hypotheses, it becomes extremely difficult to draw any substantial conclusions about the genetic foundations of language. For instance, the gene *SRGAP2* has often been invoked in discussions of language since it has been shown to be involved in cortical growth (Hillert, 2015). The occurrence of certain hominins correlates with copies of the genes, but also with the appearance of different artifacts, and so it is difficult to even generate any inferences let alone adjudicate between different hypotheses.

More broadly, Fisher (2013) makes the crucial point that genes do not specify behavioral outputs, and do not even code for specific cognitive “modules.” Rather, gene products (usually proteins) interact with one another in complex networks to construct neural circuitry through modulating neuronal proliferation and migration, neurite outgrowth, axon pathfinding, synaptic strength, and so forth. Most genes, in particular regulatory genes, play multiple roles within an organism (“pleiotropy”). In short, genes do not code for “language” or “speech,” and an individual gene is rarely expressed in only one part of the central nervous system, with *FOXP2*, for instance, being expressed in the cortex, basal ganglia, thalamus and cerebellum (Lai et al., 2003).

## Globularity

Pushing our timeline back even further now, the human lineage began around 6 mya, when our common ancestor with chimpanzees split into separate lineages. Likely the closest we have to a last common ancestor was *Ardipithecus*, who lived in trees but was capable of bipedalism. Standing at 4 feet tall, their brains are estimated to have been at around 500 cubic centimeters. The oldest fossils ascribed to the genus *Homo* (emerging around 2.5 mya) are from Kenya, Ethiopia, Tanzania, and South Africa, and include cranial and postcranial specimens. These are classified as *Homo erectus*. While there is some controversy about the earliest suggestive evidence of *Homo* in species such as *Homo habilis*, *Homo naledi*, and *H. erectus* (a fragmentary upper jaw with a partial dentition from Ethiopia, dated at 2.33 mya), these cranial and postcranial specimens are the earliest fossils we can ascribe with confidence.

Skulls of subsequent members of *Homo* exhibit an increasingly high and globular morphology, forming the marked parietal bone eminences of anatomically modern humans. With respect to the development of the posterior inferior frontal gyrus, the general trend throughout hominin evolution appears to be a reduction in size on the left relative to the right, while the region more broadly projects more laterally and antero-posteriorly on the right side. Consequently, left Broca’s area appears more globular (Balzeau et al., 2014). Recent re-evaluations of the fossil record have revealed a more complex picture of frontal lobe evolution than is typically assumed, such that the inferior frontal gyrus and Broca’s cap have indeed assumed a more globular shape (in line with the rest of the forebrain more generally), i.e., they have assumed a rounder shape as opposed to a flatter projection across the cortex (Beaudet, 2017).

Building on these concerns of globularity, a recent review of 20 *Homo sapiens* endocasts from different time periods employing computed tomographic scans and geometric morphometric analyses was conducted by Neubauer et al. (2018). Endocasts approximate outer brain morphology very closely due to the fact that the brain, meninges and cranial bones interact during development. The authors showed that while modern human brain size was assumed as early as 300 kya (hominin fossils from Jebel Irhoud, Morocco), it was not until 130–35 kya that our modern, globularised brain shape emerged (that is to say, the Jebel Irhoud fossils were not globular). Crucially, Neubauer et al. (2018) note that this process “paralleled the emergence of behavioral modernity as seen from the archeological record.” They add that “the ‘human revolution’ just marks the point in time when gradual changes reach full modern behavior and morphology and does not represent a rapid evolutionary event related to only one important genetic change” (see also Murphy, 2018 for a proposal that this process of globularisation granted “traveling” neural oscillations the ability to migrate across new areas of the cortex and subcortex).

This suggests that while the capacity for constructing hierarchically organized linguistic structures (or phrase structure building) was available before the final stages of globularisation, these documented changes in brain shape (and their concomitant neural re-wiring) likely allowed this computational system to gradually interface with other previously encapsulated cognitive systems, due to this re-shaping reducing the number of “spatial inequalities” (Salami et al., 2003) in the brain, and hence the number of possible cross-regional connections. The phrase structure capacity may have emerged first, but also may not have achieved its full, modern reach until globularisation occurred. This suggests that language-music, language-mathematics and language-morality interfaces (assuming a common computational link between these capacities, à la Hauser and Watumull, 2017) emerged at different evolutionary timepoints and that it may be possible to plot a timeline for the emergence of these interfaces. For instance, we can date musical instruments to around 35 kya (such as bone and ivory flutes; Conard et al., 2009). In terms of their neuroanatomy, mathematical knowledge and language appear to involve distinct cortical networks (Amalric and Dehaene, 2019).

Additionally, no CT data of the Herto skull (160 kya) is available, and so it is possible that modern human-like globularisation was found as early as 160 kya, possibly before. If this is the case, then a more saltationist model of language evolution may be accurate such that language interfaced with other cognitive systems rapidly. Regardless, what can be said with certainty is that modern humans exhibit a more acute basicranial angle than other Great Apes, achieving a much more extreme level of globularity.

Turning to the related theme of neurolinguistics, neuronal networks have been shown to obey Rent’s rule (a “third factor” in language evolution), a rule from computing logic, exhibiting hierarchical modularity that optimizes a trade-off between physical cost and topological complexity, such that these networks are cost-efficiently wired. Rent’s rule states that the following relationship exists between several chip parameters.

$$T=AK{}^{P}$$

where *T* is the number of terminals, *K* the number of blocks within the chip, *A* the average number of terminals for one block, and *^P^* the Rent exponent. As Sengupta et al. (2013) summarize: “A modular design balances the savings in metabolic costs, while preserving computational capacities.” A more globular braincase hosting a “folded” brain (which, through gyrification, permits a large surface area to fit inside a smaller skull), in conjunction with Rent’s rule, maximizes computational efficiency and large-scale circuit integration. The implications for cognitive evolution may be substantial.

Although these empirical discoveries are novel, the general themes supporting them remain classical. Ever since Broca (1861) and Dax (1863), human brain asymmetries have been documented, often being used to help distinguish between different species. However, the oft-discussed process of lateralisation is “probably shared by all hominins” (Balzeau et al., 2014, p. 126), and so some other neural changes may have likely been responsible for language evolution. Consider Australopithecines, who comprise the human clade along with the extant genus *Homo*. These are assumed to lack the diverse behavioral and biological features exhibited by *Homo*, though the fossil record is far from complete. The oldest stone tools have been dated to around 2.6 mya, close to the likely appearance of the first *Homo*. This had led some to speculate whether the larger brains associated with early *Homo* specimens were required for the conceptualisation involved in using this type of tool (see Mann, 2011). Indeed, throughout the evolution of *Homo* brain size has almost tripled in volume. The earliest *Homo* had a braincase volume of 510–775 cc, whereas modern *H. sapiens* exhibit braincases with volumes ranging from 1200 cc to over 1500 cc. Influences of changing climate, environmental demands, and social competition are thought to be the major influences driving brain size change (Bailey and Geary, 2009). Although the trend toward brain size increase has been well documented in hominin evolution (Sousa and Wood, 2007), there are some important exceptions such as *Homo floresiensis* (Kubo et al., 2013) and the size reduction in *H. sapiens* since the Upper Paleolithic (Balzeau et al., 2014), a period lasting from 40 to 12 kya.

## Tool Use

Another domain with implications for language evolution, and one which has long been seen as relevant not just to linguistics but cognitive science more generally, is tool use. Archeologists studying the Paleolithic period have discovered the types of technology created by *Homo*. One such technology is composed of three types of basic stone tools: hammers, cores, and flakes. These are termed Oldowan tools, or Mode 1. These tools display substantial spatio-temporal uniformity with few modifications for more than 1 million years. Hominins used Mode 1 tools to kill and butcher medium- and large-sized mammals. Stone tools were also used to access bone marrow, and the surfaces of certain tools suggest that roots might also have been pounded (Wrangham, 2009). Upon the emergence of *H. erectus*, Acheulean tools (Mode 2) emerged, which were effectively enhanced versions of Mode 1 tools with the addition of a “biface”; namely, the margins of the tools were trimmed to either produce symmetrically sharp sides (as in the Acheulean hand ax) or a modified side meeting an unmodified side (as in cleavers). Acheulean tools were used to slice open animal skins, carve meat and break bones. Two examples of cutting tools typical of early Acheulean sites are pointed hand axes and picks, involving intentional shaping. Moreover, the intentional procurement of raw materials and the development of a multicomponent quarrying process was required to produce these tools. Mode 1 tools had no existence outside their conditions of use, but Mode 2 acquired a somewhat more abstract function. *H. erectus* carried them around, using them for distinct purposes and to achieve different goals, participating in the cultural life of the species. In this sense they acquired a symbolic, memetic existence, and they also naturally helped *H. erectus* consume the necessary amounts of meat needed to fuel its enlarged brain.

Indeed, it has often been suggested that because remains of one-million-year old campfires have been discovered and are thought to have been constructed by *H. erectus*, the invention of cooking might have provided a new range of nutrients feeding brain growth in *Homo* (Aboitiz, 2017, p. 452). DeCasien et al. (2017) provide novel statistical techniques to demonstrate that primate brain size is predicted by diet, not by degree of sociality, suggesting that studies of language evolution could benefit from a shift of attention toward ecological factors. The enlarged brain, in turn, seems to have been capable of coordinating spatial representations with shape recognition, necessary requirements for a biface; a process demanding an enlarged working memory for *H. erectus* relative to earlier *Homo* (Gibson, 1993). After these advances in mentally manipulating three-dimensional Euclidean space, it is not at all clear whether substantial advances in spatial cognition were made until the present, or whether the spatial reasoning skills of modern humans are closely comparable to those of *H. erectus*. One of the less controversial topics in human evolution involves the usefulness of dietary changes in providing the necessary nutrients and energy for sustaining hominin brain enlargement in early *Homo*. The modern human adult brain uses 20% of the body’s metabolic energy, whereas new-borns use around 60% (Aiello et al., 2001), with growing brains needing a substantial range of foods (captured via sophisticated hunting tools) with high nutrient density. It is possible that these dietary (and, hence, social) changes had a selectional impact on certain aspects of speech or babbling (DeCasien et al., 2017).

Turning to Neanderthals, this species has always suffered from something of an image problem: In the early 20th century, the discovery of a Neanderthal skeleton from La Chapelle-aux-Saints in France exposed deformities which were at the time thought to be indicative of their cognitive and cultural degeneracy, yet it is now known that these were simply a reflection of the old age of the particular individual. The Neanderthals in Eurasia were a population whose lineage split from that of *H. sapiens* around 500 kya, and who disappear from archeological history around 30 kya. They exhibited use of Mode 3 tools, namely Mode 2 tools with “flake technology,” producing intricate grooves along the sides of objects (surpassed only by Mode 4, or Solutrean tools; thin, sharp blades used by modern *H. sapiens*). They also introduced hafting of stone points onto spears, and lived in small communities, enjoying little to no contact with other Neanderthal groups outside local territory. Neanderthal remains have been found across Europe, and consequently play a major role in discussions of human evolution given that both species appear to have trekked out of Africa. Relative to modern humans, Neanderthals possessed a low, flat braincase, sloping foreheads and large brow ridges. Their brains were slightly larger than those of humans. Their chests were barrel-like, indicating “a body morphology adapted to the cold conditions of ice age Europe” (Mann, 2011, p. 279). Different Neanderthal groups exhibited distinctive features: “Fossil finds in northern Israel, such as those from the Tabun and Amud caves and the skeleton lacking a skull from the Kebara cave … possess features similar to other Israeli specimens, the Qafzeh and Skhul samples, which have been termed early modern humans” (Mann, 2011, p. 280).

Neanderthals also appear to have been capable of pyrotechnology. Early Neanderthals from the late Middle Pleistocene site of Poggetti Vecchi, Italy, seem able to have appropriately selected timber to create “digging sticks” (Aranguren et al., 2018; see also Hoffecker, 2018 for a review of Neanderthal technology). Kibblewhite et al. (2015) even propose a predictive framework for the preservation of materials (including bones, teeth, metals and organic materials) in soil across the European Union based on the chemical properties of discovered materials and the soil they were found in, allowing them to predict the most likely “hot spots” for future discoveries relevant for cultural/cognitive research.

Moving forward to the time of modern *H. sapiens*, the stone tools found at the Nubian Complex in the Dhofar region of Oman have been dated at 106 kya (Rose et al., 2011), providing evidence for the existence of a northeast African Middle Stone Age technocomplex exhibiting the Levallois technique of stone knapping, a complex method involving the extraction of a small plane from a larger surface. Humans may well have been responsible for this, and if so they likely left Africa as early as 110 kya.

However, Armitage et al. (2011) document how Levallois assemblages from Jebel Faya in the United Arab Emirates share close affinities with late Middle Stone Age assemblages from North East Africa. The authors date these Jebel Faya assemblages to 125 kya, pushing the migration out of Africa even further back to around 130 kya. In addition, the Lunadong hominin fossils discovered at Luna Cave in Guangxi, southern China, include one left upper second molar (M2) and one right lower second molar (m2). Bae et al. (2014) note that M2 is exclusively assigned to modern humans, while m2 is also likely to be. The teeth are dated between 127 and 70 kya, in turn suggesting an early migration from Africa and Arabia. Bae et al. (2017) review recent results from hominin paleontology, geochronology and genetics, concluding that there must have been multiple dispersals from Africa into Eurasia, rather than a single exodus.

In summary, we can say with some confidence that the apparently human-unique capacity for language-specific syntax emerged within the last 200 kya, and we can say this thanks to the development of sophisticated tools, cultural artifacts, complex trading relationships, and paintings. Indeed Miyagawa et al. (2018) draw a connection between cave paintings and “archeoacoustics,” noting that cave art is typically connected to the acoustic properties of the chambers they are located in. Being sensitive to the echoes generated in these chambers, Miyagawa et al. speculate that cave paintings may have been a form of cross-modality information transfer through which acoustic signals are transformed into visual representations. Although we will likely never know whether these complex cave paintings demanded the existence of language to produce, they are nevertheless part of a wider movement in cultural flourishing which are indicative of substantial cognitive advances.

Given the hunter-gatherer culture in which this capacity emerged, what can we say of the “first words” (or units of semantic communication) which would have been externalized? Naturally we can only speculate, but it seems reasonable to assume that these words took the form of mimetic gestures or even sounds imitating whatever the shared object of attention was (likely food/carcasses or tools). As Studdert-Kennedy and Terrace (2017, p. 121) speculate, “[t]he vocal modality would have come to prevail, leaving hands and eyes free to go about their more important functions.” Before processes such as grammaticalization took control of complex morphology, initial vocalizations would have been simple linearizations relying on pragmatic procedures to derive the full meaning of expressions (Murphy, 2016b). Yet Cataldo et al. (2018) conducted the first assessment comparing the efficiency of speech (unaided by gesture) with gesture and also gesture-plus-speech as tool-making transmission aids. They demonstrated that subjects instructed by speech alone underperformed in stone tool-making compared to subjects instructed through either gesture alone or gesture-plus-speech. They conclude that “gesture was likely to be selected over speech as a teaching aid in the earliest hominin tool-makers,” and that “speech could not have replaced gesturing as a tool-making teaching aid in later hominins, possibly explaining the functional retention of gesturing in the full language of modern humans.” They also suggest that speech may therefore have emerged for reasons unrelated to tool-making; it may have been a response to increased trade and more complex intra-group interactions bolstered by population increases.

In 1949, one of the most influential paleontologists of the twentieth century, Simpson (1949, 291–292), wrote:

Man arose as a result of the operation of organic evolution and his being and activities are also materialistic, but the human species has properties unique to itself among all forms of life, superadded to the properties unique to life among all forms of matter and of action. Man’s intellectual, social, and spiritual natures are altogether exceptional among animals in degree, but they arose by organic evolution.

It is common in the field for researchers to claim that because language is such a complex system – “altogether exceptional” (Corballis, 2017) – its evolutionary roots must extend very far back. As DeSalle and Tattersall (2017, p. 6) review, the first anatomical *Homo* exhibited “little if any of the zeal for change and innovation, and none of the ability to reconceptualise the world, that so richly characterize their modern language-endowed descendants.” But these debates presuppose a clear understanding of what *language evolution* is, as distinct from the evolution of closely related capacities. When it comes to the relevance of the fossil record to questions of *speech evolution*, Wood and Bauernfeind (2011, p. 271) conclude their data review by claiming that “the fossil evidence for archaic hominins contains little, or no, reliable evidence about the speech capabilities of these taxa.” But, going beyond fossils, what about the evolution of language and communication, distinct from speech? Assuming, as is commonly done, some form of relationship between symbolic communication and linguistic competence, there are a number of higher cognitive capacities that we share with our close relatives according to existing paleoanthropological accounts. Consider the Makapansgat manuport, a small stone (2 × 3 inches) found amongst Acheulean tools in South Africa in 1925 and putatively collected by *Australopithecus africanus* around 3 mya (other Acheulean tools are dated somewhat later). It seems to closely resemble a human face, suggesting that Australopithecus could grasp connections between arbitrary symbolic forms and abstract meanings; otherwise known as iconicity. Since this semantic property appears so deeply rooted in hominin evolution, this might explain its prevalence amongst early religionists (see also Peterson, 1999, 2018).

Examining the neural basis of primitive tool technology, Hecht et al. (2015) compared brain responses while learning either the basic Oldowan technique or the more complex Acheulean technique. The latter exhibited increased activation in the right inferior frontal gyrus and bilaterally in other regions, suggesting an increase in the requirement for cognitive control. Toolmaking typically involves the dominant hand making repetitive, rhythmic motions while the subordinate hand holds the object and occasionally rotates it (Uomini and Meyer, 2013). According to Uomini and Meyer (2013), hemispheric dominance arose due to the separation of competing neural processing strategies, one implicated in complex sequential behaviors like hand motions, and the other involved in coarse motor routines. Coordinating two different processes simultaneously (low-frequency and high-frequency motor commands) in what can arguably be described as a hierarchically organized form of behavior (though of limited hierarchy; Stout and Chaminade, 2012) may well have led to the selection for certain neural subroutines which the language system recruited when structuring the processing of units of different hierarchical complexity, i.e., when processing multiple syllables into a single word, and ultimately processing multiple words into a single phrase. Indeed, Morgan et al. (2015) discovered that students learned to make stone tools faster under verbal instruction, pointing to a potential co-evolution between toolmaking and speech (although it should be stressed that simply because verbal instruction enhances performance on a certain task, it does not follow that verbal abilities and this given task co-evolved). Note that this hypothesis does not lead to any causal explanation for language evolution (e.g., it does not commit one to the assumption that language evolved directly from toolmaking), it simply proposes that when the language faculty did emerge it was embedded within a sophisticated computational network.

Another related example comes from the Erfoud manuport, dated at around 300,000 years old and discovered in eastern Morocco. Seemingly collected by *H. erectus*, the manuport is a cuttlefish bone shaped like a phallus (Everett, 2017). What is the possible relationship of these findings to language evolution? Conceiving of language as a recursive combinatorial system involving the construction of hierarchically organized syntactic objects, generative linguists such as Hornstein (2009) or Chomsky (2010) would likely not be too impressed with a penis-shaped cuttlefish bone. Yet clearly the capacity to bind bodily concepts either to concrete instantiations or more abstract symbolic representations in the form of manuports involves some form of impressive semantic mapping of the kind subsequently exploited by the language system in anatomically modern humans. Moreover, the development of the 300–400,000-year-old Schöningen spears point toward a sophisticated culture amongst *Homo heidelbergensis*, since not only do they act as tools but they also have symbolic cultural meaning, such that the spear can denote the act of hunting in abstraction, i.e., in the absence of any particular hunt. And unlike many other tools used throughout the animal kingdom, Everett (2017, p. 143) notes that these spears display aspects of Peircean signs in that “only certain parts of the tools are meaningfully connected to their tasks, e.g., the edge of the tool.” This greater degree of abstraction seemingly came about shortly before the time that language would have emerged among anatomically modern humans (300–200 kya), and so the generous and rapidly developing cognitive toolbox of *H. heidelbergensis* (a variant of *H. erectus*, or even identical according to some researchers) may well have been passed down to modern humans. *H. heidelbergensis* additionally had a great number of nerves linking the brain and tongue than its predecessors, suggesting that it possessed the ability to refine and control vocalizations.

With this toolbox at the ready, the bow and arrow was used by humans as early as 71 kya (McBrearty, 2012), a weapon which goes considerably beyond the complexity of the spear, likely involving a degree of sophisticated communication in order for it to be taught and implemented in a coordinated, strategic fashion. Likewise, most researchers concur that the capacity for complex symbolic thought (i.e., combining distinct symbolic representations in novel, “imaginative” ways, of the kind found in polysemy; Pustejovsky, 1995, 2008; Falkum and Vicente, 2015; Murphy, 2019b) was needed to construct bodily ornaments such as beads and decorative objects (Vanhaeren et al., 2006; Texier et al., 2010); both of which appeared around 100–60 kya.

The capacity for complex orthography, and potentially also the ability to associate symbolic meaning with indentations, can also be found as far back as 540 kya in the form of zigzag marks on a shell made by a member of *H. erectus* and found in Java. Interestingly, a sea voyage was likely made by the creator (from mainland Asia to Java), who might have represented the sea through these patterns. The intentional act of creating marks to represent abstract icons also provided an important pre-linguistic trait for anatomically modern humans, who presumably would have been able to externalize their new Language of Thought after the emergence of human-specific syntax in precisely the same way as *H. erectus*, with the exception of using such markings to represent more complex, composite representations, as opposed to simple concepts like SEA or FACE. Likewise, *H. erectus* crafted a wide number of tools (including choppers and pounders). These could not have feasibly been created systematically from any random motor sequence, but require planning and imagination, as well as the ability to communicate to others the methods of production. The expanded cognitive power required for mastering these procedures, which soon became a necessary part of survival (in particular in the event of tribal warfare), may well have led to an important role for natural selection: namely, selection for expanded fronto-parietal circuits to satisfy the growing demand for cognitive control networks. Thus, we find the world’s oldest piece of art, the 250 kya Venus of Berekhat Ram, a rock carved in a female shape with evidence of intentional red ochre coloring for decoration, an object crafted with precision and imagination.

These ideas – of syntax ultimately being couched within pre-existing semantic properties – are quite distinct from the hypothesis proposed by Everett (2017). His claim is that “with symbols + concatenation, there is language” (2017, p. 160). While a certain amount of compositionality might be derived from a semantic system relying on this architecture, hierarchically organized phrases plus long-distance dependencies cannot emerge from this. Combining representations of any format into syntactically hierarchical phrases is not a job for symbolism and concatenation alone (Murphy, 2015, 2016a). Likewise, the engraved ochre and bones found in Blombos Cave are suggestive of symbolic manipulations, yet as Botha (2011, p. 307) notes any links to syntactic language are highly questionable since “beads, ochres, and engraved bones cannot stand as evidence for modern cognition, including language, unless it is specified what cognitive abilities these artifacts require.” Indeed, although the use of pigments pre-dates Blombos Cave and even implicates Neanderthals, these were non-symbolic and displayed little variation (Neanderthal pigments were generally black, for instance).

Finally, one of the core characteristics of the tools of early *H. sapiens* is that they were crafted for durability just as much as immediate usefulness. This suggests a familiarity not only with symbolic behavior, but with long-range planning. These planning and strategizing capabilities are neurologically and computationally separate from purely linguistic processes, suggesting that modern cognition demanded certain developments in executive reasoning skills as well as the evolution of language.

The general picture that emerges here is the following: The Oldowan tools dated around 3 mya are suggestive of dexterity, motor control and intentional modifications of inanimate objects; the Acheulean tools dated slightly later (perhaps around 2 mya) are suggested of hierarchical cognition and/or complex motor planning, along with complex emotions. The axes, cleaver and spears of *H. heidelbergensis* dated around 400 kya are suggestive of visual imagination, emotional control, symbolism, and possibly a sense of self. The Levallois method is generally dated around 300 kya, and is suggestive of advanced hierarchical cognition, tuition, and an unusual degree of patience. Lastly, the technology of modern *H. sapiens* dated around 200 kya is suggestive of an improved memory, creativity, and an awareness of past and future.

## Domestication

Closely tied to the theme of language evolution is the broader, and related (indeed, arguably identical) theme of *human evolution*. If we define *H. sapiens* based on derived skeletal features, then the fossil record would place human origins somewhere in the African late middle Pleistocene. The relevant fossil data includes Omo Kibish 1 and the Levantine material from Skhul and Qafzeh. Some of the oldest morphologically modern humans have been found at the Omo Kibish sites, and date to ∼195 kya (McDougall et al., 2005). Yet the genetic data indicates that both anatomically modern humans and *Homo neanderthalensis* shared a common ancestor in the middle Pleistocene (400–700 kya), a date some 200 kya earlier than the fossil-determined date.

Stringer (2016) notes that findings of this kind suggest that the morphology of *sapiens* exhibited no linear progression, and “there was chronological overlap between different ‘archaic’ and ‘modern’ morphs” (2016, p. 1). Extant humans exhibit a number of shared traits, including a high neurocranium, a small face retracted under the frontal bone, small discontinuous supraorbital tori, and a narrow trunk and pelvis (Stringer, 2016). Anatomically speaking, it is possible to detect humans in the fossil record through focusing on these and broader features like cranial globularity and basicranial flexion (Arsuaga et al., 2015). Particularly relevant for language is a certain feature of the cranial vault: The parietal region is highly distinctive in humans, being expanded in certain areas (Bruner, 2010). Modulating and strengthening the connections of this expanded parietal region with other regions, such as anterior temporal regions and subcortical structures like the thalamus, may have contributed to novel cross-modular communication.

In this connection, it is increasingly becoming clear that the topic of *domestication* has clear potential to inform our understanding of human brain evolution. The notion that anatomically modern humans are a fundamentally domesticated species has a long and rich history, dating back to Darwin (1871) and Boas (1938), with the latter commenting that “[m]an is not a wild form, but must be compared to the domesticated animals. He is a self-domesticated being” (Boas, 1938, p. 76). Concerning the general processes of self-domestication, Boas added that “[i]t is likely that changes of mental character go hand in hand with them” (1938, p. 140), and it is only very recently that researchers have been able to propose concrete hypotheses which expand on these speculations.

Domesticated species (including dogs, cats, foxes, pigs, and sheep) are usually defined based on their shared phenotypic traits, referred to collectively as the “domestication syndrome” (Zeder, 2012) and which include depigmentation, reduced ears, shorter muzzles, smaller teeth, smaller cranial capacities, and a reduction of sexual dimorphism (feminisation). Many of these features are exhibited by anatomically modern humans, and in fact distinguish humans from Neanderthals (Theofanopoulou et al., 2017), and they may also reflect a generalized deficit in the neural crest, an embryonic structure responsible for pigmentation and the cranial skeleton, amongst other things (Wilkins et al., 2014). Domesticated animals used to be regarded as entirely separate species but are now thought of as sub-species of their wild progenitors. Le Douarin (1980) discovered that transplanting neural crest cells from chicks to quails resulted in the chimeric hatchlings producing intermediate chick/quail vocalizations, suggesting that the process of self-domestication, involving the neural crest, contributed in some fashion to the emergence of vocal learning. Interestingly, Theofanopoulou et al. (2017, p. 4) document how interspecific domestication events suggest that “the selective pressure for our self-domestication need not have been qualitatively different from those experienced by other species.” For instance, the silver fox (*Vulpes vulples*) was intentionally domesticated through a project initiated by Belyaev (1979) based on a single criterion: tameness toward humans. After only 20 years of selection for tameness, a range of features typically associated with domestication emerged, suggesting a strong, causal link between the above noted phenotypic characteristics of domesticants.

It is therefore likely that selection for tameness, prosocial behavior or related traits associated with the syndrome brought about human self-domestication after the split from our last common ancestor. Self-domestication can potentially explain – “for free” – a number of human-specific traits, with the possible exception of the descended larynx, an explanation for which remains in relative obscurity. Speaking to this hypothesis, recent work suggests that humans, unlike monkeys, are adept at turning competitive situations into cooperative ones (Marquez, 2017). Tomasello et al. (2005, p. 685), discussing “shared intentionality,” note that “it is almost unimaginable that two chimpanzees might spontaneously do something as simple as carry something together or help each other make a tool.” More generally, as Theofanopoulou et al. (2017, p. 12) note: “It is also not unreasonable to suspect that byproducts of the domestication process, such as enhanced sensory-motor perceptual and learning pathways, may provide a foundation for more complex communicative abilities, including vocal learning abilities.”

Recent work has emphasized the potential for studies of dog vocal social perception to enhance our understanding of how linguistic and non-linguistic signals are represented in the mammalian brain in particular given that dogs have lived in anthropogenic environments from at least 32–16 kya (Andics and Miklósi, 2018). This perspective goes somewhat beyond the standard focus on great apes, giving the study of vocal social perception a broader mammalian basis. It has been argued in the literature that dog domestication enabled this species to survive in small human groups (Serpell, 1995), fast becoming man’s “best friend,” with this process selecting for dogs with the genetic potential to develop human-compatible behaviors. Dog brains also appear to have dedicated voice areas, preferring conspecific vocalizations over other sounds (Andics et al., 2014). These areas are located in anterior temporal regions, including the bilateral temporal poles. One possible interpretation of these findings, as Andics and Miklósi (2018, p. 60) note, is that “conspecific preference in dogs and humans relies on homologous brain structures, implying that voice areas have been there in the last common ancestor of the two species, but convergent evolution provides an alternative interpretation that voice areas developed independently in the ancestors of dogs and humans, after their lineages split.”

The importance of examining the brain in order to properly distinguish humans from Neanderthals is highlighted in recent work in paleoneurology. Mounier et al. (2016) document how endocranial features are more informative than features of the calvarium (supporting research efforts geared toward domestication) and how human endocranial anatomy dramatically changed during the end of the Middle Pleistocene. Cultural development seems to have appeared alongside domesticated features like a smaller braincase, with a reorganization of the cranium altering many neural features.

Wrangham (2009) maintains that the cultural developments of anatomically modern humans are the result of self-domestication via inhibiting aggression and related traits. His line of research points to comparable developments within certain ape societies. For example, while chimpanzees display a range of cooperative traits their culture is typically plagued by aggression and violence (Hare et al., 2012). Bonobos (pygmy chimpanzees), in contrast, display a juvenile appearance (in line with domestication models) and live in far more peaceful societies (though, it should be noted, not as peaceful as stereotypes would suggest due to clear carnivorous tendencies). Like humans, bonobo societies are much larger than those of chimpanzees, with the rapidly increasing size of early human tribes likely playing a role in their domestication. As Aboitiz (2017, p. 452) summarizes: “As we domesticated other species, we adapted ourselves to the process of domestication, forming an evolutionary circle that maintained our genetic evolution and drags other species with it.” This cyclic process of self-domestication involved adapting to the needs of human groups while also domesticating a range of plants and animals in ways dynamically responding to such needs, with the newly domesticated plants and animals in turn influencing the social structure of human societies (see also Murphy, 2019a).

Turning to a related field of study, Okanoya (2012, 2013) reports that comparisons of the songs of wild finches (white-rumped munia) and domesticated finches (Bengalese finch) suggest that the latter produced songs of greater complexity, differing in acoustical morphology and the order of elements. Lansverk et al. (2018) replicate and expand on these results and also explore their genetic underpinnings. The sound density was also found to be 14 dB higher in Bengalese finches than in white-rumped munias during recordings from identical settings. The most recent research in this direction has even suggested that domesticated birds have smaller brains but a larger cortex, in particular the forebrain (Olkowicz et al., 2016). As such, domestication seems broadly responsible for increases in syntactic complexity, with the complex syntax of Bengalese finch songs developing from simple neurological changes (Katahira et al., 2013).

In summary, it appears from recent evidence that self-domestication helped lay the groundwork for enhancing in modern humans some of the communicative, semantic and syntactic capacities of our ape ancestors.

## The Cerebellum and Speech

Although left-frontal and parietal regions enjoy the most attention in discussions of language evolution, I would like to briefly address the potential importance of the *cerebellum*, which is increasingly being implicated in language processing. Of course, there are many other regions in the brain for which the same type of evidence presented below could be used in support of the idea that they are important for language, but the cerebellum more tightly fits into the present theme of brain shape modification.

The human cerebral cortex is approximately 3 millimeters in depth, while the cerebellum is considerably larger and contains 60 out of the brain’s 86 billion neurons. Yet its role in higher cognition remains somewhat unclear. Pursuing the above line of inquiry, Ogihara et al. (2018) conducted a three-dimensional geometric morphometric analysis of reconstructed Neanderthal and early human endocasts. Their results indicated that ecto- and endocranial shapes are quantitatively different between the two species. The cranium of early humans displayed relative enlargement of the cerebellar region and a notable parietal expansion. This is perhaps the strongest evidence that the neuroanatomical organization of the two species was significantly distinct. Following directly on from this documented cerebellum expansion, Tanabe et al. (2018) note that while the cerebellum has typically been seen as being involved largely in fine motor control, an emerging consensus is that this region is also involved in certain cognitive functions, including language. It exhibits a unique gross anatomy and microstructure, and the cerebellar cortex contains circuitry functioning as a learning system able to construct and store internal models of the world. Tanabe et al. (2018) show that the greater volume of the cerebellar cortex, the greater number of internal models it is able to construct and store. It seems likely that the cerebellum is therefore implicated in forms of long-term memory, with some of the complex representations it stores being constructed initially by the language system. In this sense, it may act as a post-linguistic long-term storage site, functionally distinct from parts of Broca’s area (e.g., BA 44 v, following standard sub-parcellation) which seem to act as a short-term memory “buffer” site for phrase structures. Finally, cerebellar dysfunctions in humans lead to distinct speech motor deficits referred to as ataxic dysarthria (Ackermann, 2008; see also Murphy and Benítez-Burraco, 2017). The cerebellum is assumed to be involved in the control of coarticulation effects given its involvement in sequencing syllables into fast, rhythmically structured larger utterances. Nozaradan et al. (2017) also provide EEG evidence that the cerebellum and basal ganglia are involved in the neural representations of rhythmic sequences, in particular those demanding the encoding of precise sub-second events (see also Obleser et al., 2017).

More recently, Smaers et al. (2018) investigated the lateral cerebellum (a structure unique to mammals) across a range of species and mapped its evolutionary diversification, finding that relative volumetric changes of the lateral cerebellar hemispheres are correlated with measures of domain-general cognition in primates. These are furthermore characterized by a combination of parallel and convergent shifts toward similar levels of expansion in distantly related mammalian lineages. This suggests that increased behavioral complexity (for our purposes, of the kind found in the emergence of language) from a range of directions may be traced back to a common selection on a shared neural system, the cerebellum. This implies that this brain region aided certain other forms of higher cognition in a range of mammals, while in humans it seems to have aided rhythmicity and memory load, directly exploited by the language system.

Deepening these connections, Pidoux et al. (2018) show that the cerebellum provides a strong input to the song-related basal ganglia nucleus in zebra finches. Cerebellar signals are transmitted to the basal ganglia via a disynaptic connection through the thalamus, before being conveyed to their cortical target and to the premotor nucleus controlling song production. These authors also showed that cerebellar lesions impair juvenile song learning.

As such, paleoneurological evidence bearing on the morphology of the cerebellum will likely inform our understanding of when certain language-related capacities emerged.

## Future Directions

The unanswered questions emerging from this discussion cut across a range of domains: Which features of (self-)domestication have had an impact on the language system architecture? How does the speed of the molecular clock impact either saltationist or adaptationist hypotheses concerning the emergence of language? What are the potential ways domestication can influence the externalization component of a given species? Which factors (e.g., nutrition, climate) had the potential to impact features of human cognition relevant to language comprehension during the course of modern human evolution? To what extent could future studies of archaic hominin admixture provide insights into the evolution of language? What specific brain regions were impacted by globularisation, and how did this process impact language (and language-related) processes? How might globularisation have impacted higher cognition in other species?

## Author Contributions

The author confirms being the sole contributor of this work and has approved it for publication.

## Conflict of Interest Statement

The author declares that the research was conducted in the absence of any commercial or financial relationships that could be construed as a potential conflict of interest.
